# Stress Response and Virulence Potential Modulating Effect of Peppermint Essential Oil in* Campylobacter jejuni*

**DOI:** 10.1155/2019/2971741

**Published:** 2019-01-03

**Authors:** J. K. Kovács, P. Felső, Gy. Horváth, J. Schmidt, Á. Dorn, H. Ábrahám, A. Cox, L. Márk, L. Emődy, T. Kovács, Gy. Schneider

**Affiliations:** ^1^Department of Medical Microbiology and Immunology, University of Pécs Medical School, Hungary; ^2^Department of Pharmacognosy, University of Pécs Medical School, Hungary; ^3^Department of Biochemistry and Medical Chemistry, University of Pécs Medical School, Hungary; ^4^Department of Medical Biology and Central Electron Microscope Laboratory, University of Pécs Medical School, Hungary; ^5^Department of Biotechnology, Nanophagetherapy Center, Enviroinvest Corporation, Pécs, Hungary; ^6^Veterinary Medical Research Institute, Hungarian Academy of Sciences, Budapest, Hungary

## Abstract

*Campylobacter jejuni* is one of the most common food-borne bacteria that causes gastrointestinal symptoms. In the present study we have investigated the molecular basis of the anti-*Campylobacter* effect of peppermint essential oil (PEO), one of the oldest EO used to treat gastrointestinal diseases. Transcriptomic, quantitative reverse transcription-polymerase chain reaction (qRT-PCR) and proteomic, two-dimensional polyacryl amid gel electrophoresis (2D-PAGE) methods have revealed that, in the presence of a sublethal concentration of PEO, the expression of several virulence-associated genes was decreased (*cheY* 0.84x;* flhB* 0.79x;* flgE* 0.205x;* cadF* 0.08x;* wlaB* 0.89x;* porA* 0.25x;* cbf2* 4.3x) while impaired motility was revealed with a functional analysis. Scanning electron micrographs of the exposed cells showed that, unlike in the presence of other stresses, the originally curved* C. jejuni* cells straightened upon PEO exposure. Gaining insight into the molecular background of this stress response, we have revealed that in the presence of PEO* C. jejuni* dominantly exerts a general stress response that elevates the expression of general stress genes like* dnaK*,* groEL*,* groES* (10.41x, 3.63x, and 4.77x). The most important genes* dps*,* sodB*, and* katA* involved in oxidative stress responses showed however moderate transcriptional elevations (1,58x, 1,55x, and 1,85x).

## 1. Introduction

*Campylobacter jejuni* is the most common gastrointestinal bacterial pathogen around the world [1]. This microaerophilic bacterium belongs to the intestinal flora of birds [2] and therefore human cases are mostly associated with fecal contamination during slaughter and the subsequent consumption of undercooked poultry products [3]. Details of the pathogenic process are still not entirely clear, but the unambiguous importance of motility by flagella and adhesion mediated by cell surface factors like CadF [4], PEB1 [5], and PEB4 [6] is confirmed. Although infections caused by* C. jejuni* are usually self-limiting and rarely require therapeutic intervention, the emergence of antibiotic resistance among isolates [7–9], the recent description of a hypervirulent [10], and a multidrug resistant clone [11] from animal husbandries raises major epidemiological and healthcare concerns. Therefore new strategies are needed to control this food-borne bacterium. One option for this is the application of essential oils (EOs) with a broad antimicrobial spectrum, offering an alternative opportunity for prevention [12].

Recent studies have demonstrated the potential of juniper preparations to impede adhesion [13] of* C. jejuni* to polystyrene surfaces. Thyme and olive extracts may inhibit adhesion not only to artificial surfaces, but also to intestinal epithelial cells [14]. These studies illustrate the capacity of extracts to impede survival of* C. jejuni* on solid surfaces, while others revealed the virulence potential modulating effect of clove EO using transcriptomic and phenotypic methods [15].

Peppermint (*Mentha piperita*) is one of the oldest and most highly regarded herbs for aiding digestion and treating gastrointestinal diseases [16]. This feature was clinically confirmed, along with its antispasmodic, anti-inflammatory, and antibacterial properties [17]. Although the inhibition of* C. jejuni* growth by peppermint oil has been previously reported [18, 19], no detailed study has been dedicated to the evaluation of its anti-*Campylobacter* and antivirulent effect. Environmental stresses can determine the potential of a microorganism to evoke a disease as stress responses were revealed to be major factors in virulence gene expression [20]. It is generally thought that due to membrane lesions, the EO evokes an oxidative stress response [21–23]. However,* C. jejuni* uniquely lacks the classical oxidative stress response regulatory elements SoxRS and OxyR [24] present in a wide range of bacteria. It only expresses DNA protector protein (Dps), Superoxide dismutase B (SodB), alkyl hydroperoxide reductase C (AhpC), and catalase (KatA) to combat reactive oxygen species (ROS). Recent studies have revealed that in* C. jejuni,* general and chemical stresses are managed by important molecular chaperones like GroEL [25] and DnaK [26]. The role of GroEL and DnaK was demonstrated under different stress conditions including low osmolarity medium [27], oxidative circumstances [28], low or high temperature [29, 30], and the presence of zinc-oxide [31]. These stress situations led to the transformation of* Campylobacter* into a viable, potentially pathogenic, but not culturable (VBNC), state [32–34]. This state is characterized by typical rounded cell morphology.

The main objectives of this study were (i) to confirm the antibacterial effect of PEO on a broad* Campylobacter jejuni* isolate collection, (ii) to reveal the virulence potential modulating effect of this EO, (iii) to get an insight about the characteristic changes that typify this stress response, and (iv) not least to reveal the type of the stress response with which* C. jejuni* answers this environmental challenge. For this purpose we applied phenotypic, transcriptomic, proteomic, and electron-microscopic methods.

## 2. Materials and Methods

### 2.1. Bacterial Strains, Culture Conditions, Essential Oils

Individual lawns of 190 independent local human isolates from a thoroughly characterized* C. jejuni* collection [9] and 4 reference strains (NCTC 11168, RM1221, 81-176, 81116) were screened with the drop plate method (10 mg / drop) in order to confirm the general antibacterial effect of PEO on this species. Diameters of the individual inhibition zones were measured 48 hour after incubation under microaerophilic condition at 37°C. For the detailed analyses, based on phenotypic, genomic, proteomic investigations, the well characterized reference* C. jejuni *strain NCTC 11168 was used. Logarithmic (OD_600_=0.6) cells were prepared as follows: bacteria were grown on CCDA (Charcoal Cefoperazone Deoxycholate Agar) at 37°C under microaerobic conditions (Don Whitley Scientific, United Kingdom) for 24 h. Cells were collected with a loop and suspended in PBS. Bacterial cell counts were standardized in PBS by setting the optical density (OD) to 1.0 (approx. 4x10^8^ mL^−1^ cells) at 600 nm. The suspension was then diluted 50 times in brain heart infusion (BHI) medium (starter culture) to the required volume and grown under microaerobic conditions until it reached OD_600_=0.6. The resulting logarithmic suspension served as a starting suspension for all experiments. PEO containing pure extract without solvent was purchased from AROMAX Co. (Hungary). The EO quality was consistent with the standards detailed in the European Pharmacopoeia (4th edition).

### 2.2. Determination of the Minimum Inhibitory Concentration (MIC) and Minimum Bactericidal Concentration (MBC)

MIC and MBC values of PEO were determined using the crude EO without the addition of detergents [15, 35]. OD_600_=0.6 cultures of* C. jejuni* were diluted 10 times in BHI medium, and 5 mL was aliquoted into each well of 6 well tissue culture plates. Different volumes of PEO (0.25 *μ*L, 0.5 *μ*L, 1 *μ*L, 2 *μ*L, 4 *μ*L, 8 *μ*L, 16 *μ*L, 32 *μ*L, 64 *μ*L, and 128 *μ*L) were added to give a spectrum of concentrations (0.05, 0.1, 0.2, 0.4, 0.8, 1.6, 3.2, 6.4, 12.8, and 25.6 mg mL^−1^). No PEO was added to the control wells. Samples were retrieved after 24 h incubation under microaerobic condition at 37°C, and CFUs were determined by making serial dilutions. The number of colonies was counted and recalculated to 1ml volume, and MIC was defined as the lowest concentration of PEO that inhibited visible bacterial growth. MBC was defined as the lowest concentration that killed 99.9% of the initial inoculum. Experiments were performed 3 times on 3 different days.

### 2.3. Scanning Electron Microscopy (SEM)

SEM was used to reveal morphological changes between the PEO treated and untreated* C. jejuni* cells as previously described [15]. Briefly, 100 *μ*L of PEO treated (150 *μ*g mL^−1^) and nontreated* C. jejuni* NCTC 11168 cells (OD_600_=0.6) was fixed with an equal quantity of 2.5% (v/w) glutaraldehyde (PBS, pH 7.4). After 2 h incubation at 21°C, cells were centrifuged (12,000 x g, 1 min), gradually dehydrated in ethanol (10 min in 50%, 10 min in 80%, and 10 min in 96%), and dried for 30 min first with 50% and then with 100% hexamethyldisilazane (HMDS, Fluka, USA). The resulting* C. jejuni* cells were coated with a layer of gold using fine coat ion-sputter JFC 1100 (JEOL, UK), after mounting them on aluminium stubs. For visualization, 16 kV and 10,000x magnification was used on a JSM 6300 Scanning Microscope (JEOL, UK). Changes in cell morphology were ranked: 1, typical spiral form; 2, spiral shape not definite; 3, straightened and elongated shape. Based on these criteria affected cells on 5 fields of 10,000x magnification were observed and categorized and their distributions were expressed as a percentage of the nonaffected cells. Treatments for SEM analysis were repeated once.

### 2.4. Motility Assay

Motility assay was performed in 3 parallels as described earlier [15, 35]. One *μ*l of the OD_600_=0.6 culture of* C. jejuni* NCTC 11168 was added to the middle of 0.3% BHI agar plates (20 mL) lacking or containing PEO (150 *μ*g/mL or 50 *μ*g/mL) with a standard 1 *μ*l loop (Sarstedt, Germany). Diameters (mm) of the spreading zones were determined after 24 h incubation at 37°C under microaerobic conditions.

### 2.5. Total RNA Isolation and cDNA Synthesis

Total RNA was isolated with the RNAzol kit (Molecular Research Center, USA) according to the manufacturer instructions 10 min after exposure of the cells to PEO (150 *μ*g mL^−1^). For this, 10 mL standardized (OD_600_=1) suspensions of PEO treated and nontreated cells of* C. jejuni* NCTC 11168 were centrifuged (8,000xg, 15 min) and then suspended in RNAzol. A 20 min DNase treatment (Roche, Switzerland) at 30°C was applied to remove DNA remnants. This reaction was stopped with 2 *μ*l 0.2 M EDTA for 10 min at 75°C, and the obtained RNA samples were purified by RNeasy Mini Kit (Qiagen, Germany). RNA amounts were quantified using the ND-1000 Nanodrop Spectrophotometer (Thermo Scientific, USA) and 0.2 *μ*g was used for cDNA synthesis according to the user's guide (Superscript Reverse Transcriptase III, Invitrogen, USA).

### 2.6. Quantitative Real-Time PCR (qRT-PCR) Analysis

qRT-PCR was implemented to reveal and confirm changes in the gene expression profiles of treated and untreated* C. jejuni* cells using 44 primer pairs targeting genes involved in (i) the pathogenic process, (ii) stress response, (iii) basic metabolism, and (iv) transcription regulation (Table S1.). SYBR green master mix (Bio-Rad, USA), the Rotor Gene, RG3000 apparatus (Qiagen, Germany), and the following conditions were used: 15 s at 96°C, 15 s at 50°C, and 25 s at 72°C, with 45 repeats. Melting-curve analyses were performed immediately after each amplification. Samples were normalized to the phosphoglucosamine mutase (*pgm*) gene that served as an internal standard [36]. No RNA template was present in the negative controls.

The 2^−ΔΔCT^ method [37] was used to calculate the relative* n*-fold changes of transcriptions of the examined genes between the treated and untreated samples. For this, the results of 3 independent qRT-PCR runs were obtained. Groupwise comparison and statistical analysis of the relative expression results were performed with the Relative Expression Software Tool (REST) 2009 [38].

### 2.7. Two-Dimensional Gel Electrophoresis (2D SDS-PAGE)

Preparation, separation, and analysis of the protein content of PEO treated (150 *μ*g mL^−1^) and untreated NCTC 11168* Campylobacter jejuni* cells were carried out, as previously described [15], 10 min after exposure to 150 *μ*g mL^−1^ PEO. Briefly, after sonication in 50 mM Tris-HCl, 1 mM EDTA pH 7.4, the protein concentrations were determined with DC™ Protein Assay Kit (Bio-Rad). Protein samples (100 *μ*g) were dissolved in the 2D sample buffer (8M urea, 2% CHAPS, 50mM DTT, 0.2% Bio-Lyte 3/10 ampholytes, trace bromophenol blue, all from Bio-Rad) and loaded on IPG strips (7 cm, pH 3-10, Bio-Rad) for isoelectric focusing (IEF) (1., 250V, 2 h, linear, 2., 500V, 2 h, linear, 3., 4000 V, 10000 Vh). IEF strips were equilibrated for 10 min in 6 M urea, 2% SDS, 20% glycerol, trace bromophenol blue, and 2% DTT (Bio-Rad) and then for 10 min in the same solution containing 2.5% IAA instead of DTT. Separation of proteins according to molecular mass was performed by 2D SDS-PAGE at 80 V for 20 min and 120 V (20 min), visualized by staining with coomassie R-250, and scanned (Pharos FX, Bio-Rad). For protein identification and mass spectrometric analysis the bands of interest were excised from the gels. SDS-PAGE experiments were carried out twice for both the treated and untreated samples.

The excised gels were cut into small pieces and digested as previously described [15]. 100 mM ammonium bicarbonate (Bio-Rad) was used to remove coomassie and SDS remnants from the gel slabs. Dehydration in acetonitrile was followed by 10 mM DTT (Bio-Rad) treatment in order to reduce disulfide bridges. Free-SH groups were alkylated with 55 mM iodoacetamide (Bio-Rad) solutions and these modified proteins were in-gel digested with side-chain protected trypsin (Promega, Madison, WI). Peptides were extracted from the gel, dried, and redissolved before mass spectrometry analysis. The 2D SDS-PAGE experiments were repeated twice from two different treatments (a total of 4 runs). The only spots analyzed were those that were unambiguously detected with liquid chromatography-mass spectrometry. Changes in expressions of the identified proteins were confirmed by RT-PCR.

### 2.8. Liquid Chromatography-Mass Spectrometry (LC-MS)

The Waters nanoACQUITY ultra-performance HPLC equipment coupled with a nano-ESI MS instrument (Bruker Maxis 4G UHR-QTOF) was used to analyze excised and prepared spots from the gels [30]. 5 *μ*l aliquots were injected and separated on a 1.7 *μ*m BEH130 C18 analytical column (75 *μ*m x 100 mm) using gradient elution at a flow rate of 350 nl min^−1^. Two eluents were used, A (aqueous formic acid solution: 0.1%) and B (acetonitrile/formic acid: v/v 99.9/0.1%). The scanning range was 100–3.000 m z^−1^, and nitrogen was used as nebulizer gas (0.6 bar). Conditions of drying gas flow rate were 4 l min^−1^ at 180°C, with capillary voltage set to 3.8 kV. For protein identification fragmented peptides were processed with Data Analysis 3.4 Software and identities were searched in the NCBI and the Swiss-Prot databases. Parameters were set to allow one missed cleavage site, accepting 80 ppm mass error at the MS^1^ and 0.3 Da at the MS^2^ mode.

### 2.9. Gas Chromatography-Mass Spectrometry (GC-MS)

GC-MS was implemented to identify PEO compounds using the Agilent 6890N/5973N GC-MSD (USA) system with Agilent HP-5MS capillary column (30 m × 250 *μ*m × 0.25 *μ*m). Detailed run conditions are described in detail elsewhere [15]. Peaks were identified based on their retention times and standard addition while percentage evaluation was performed by using area normalization. Compound composition of the sample was analyzed twice in two consecutive runs.

### 2.10. Thin Layer Chromatography Combined Direct Bioautography (TLC-DB)

Compound composition and antibacterially active compounds of PEO were visualized in parallel on two preconditioned (100°C for 30 min) 5 x 10 cm 60 F_254_ thin layer chromatography (TLC) plates (Merck, Germany). Prior to the experiment 100 *μ*L PEO sample was dissolved in 500 *μ*L absolute ethanol. From this solution 0.2 *μ*L aliquots were deposited in a horizontal thin line at the bottom of the plates, and ethanol was served as a solvent control. 10 mg/mL menthol, isomenthone, and menthone (Sigma, Hungary) were used as compound controls (1 *μ*L) with known running features. TLC plates were developed with toluene–ethyl acetate (95:5) in a saturated twin trough chamber (Camag, Switzerland). Separated compounds of PEO were visualized by dipping one TLC plate into the ethanolic vanillin–sulphuric acid reagent and heated for 5 min at 90°C. Separated compounds were identified based on their R_f_ values, determined by the known standards (menthol, isomenthone, menthone). The other plate was incubated for 1 h at 37°C under microaerobic conditions in 50 mL BHI-*C. jejuni* suspension (3 x 10^8^ cfu mL^−1^). Then the plates were immersed in an aqueous solution of 3-(4,5-dimethylthiazol-2-yl)-2,5-diphenyltetrazolium bromide (MTT, 0.05 g/ 90 mL) for 10 s and incubated for 2 h under microaerobic conditions. Antibacterial activities of the separated compounds were revealed as white spots against the bluish background [15]. All measurements were performed in duplicate.

### 2.11. Statistical Analysis

Statistical analysis of the relative gene expression results was performed with the Relative Expression Software Tool (REST) 2009 [38]. A simple percentage distribution was applied for morphology and motility evaluations.

## 3. Results

### 3.1. Minimum Inhibitory Concentration and Minimum Bactericidal Concentration of PEO against* C. jejuni*

**Figure 1:**
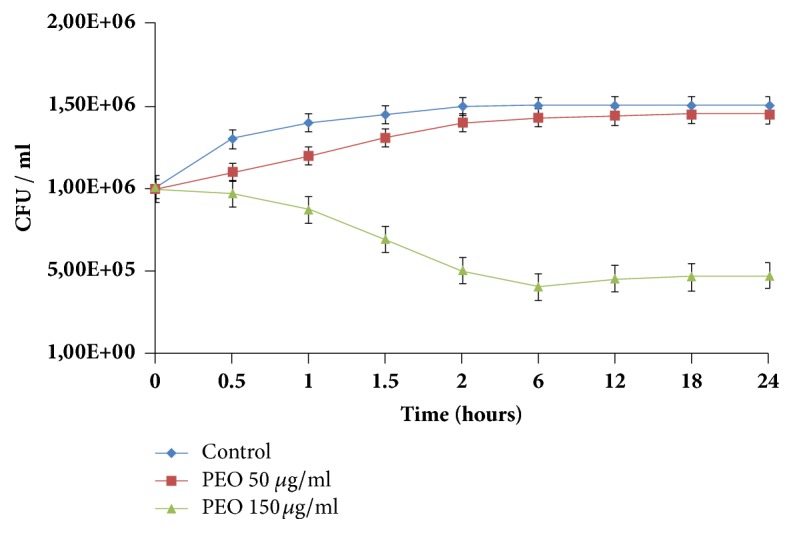
**Influence of two different peppermint essential oil (PEO) concentrations on the proliferation kinetics of* C. jejuni* strain NCTC 11168.** The applied 150 *μ*g mL^−1^ concentration of PEO was between the MIC (100 *μ*g mL^−1^) and MBC (400 *μ*g mL^−1^) values and proved to be sublethal. In contrast, the 50 *μ*g mL^−1^ concentration—used in the motility assay—was under the MIC value, having no drastic influence on proliferation.

Drop plate tests have revealed a 28-32 mm inhibition zone on the lawn of all the tested* C. jejuni* strains in the presence of 10 *μ*l PEO. No PEO resistant* C. jejuni* isolate was detected among the members of our collection. Minimum inhibitory concentration (MIC) and minimum bactericidal concentration (MBC) of PEO in the case of NCTC 11168 were 100 *μ*g mL^−1^ and 400 *μ*g mL^−1^, respectively. The 150 *μ*g mL^−1^ PEO concentration was between the MIC and MBC value that exerted a 1.5 order of magnitude loss in living bacterial cell number in 24 h on the tested NCTC 11168* C. jejuni* isolate by diminishing the living cell counts to 99.5% in 10 min and to 97% in 30 min (Figure 1).

### 3.2. Morphological Analysis of* C. jejuni*

**Figure 2:**
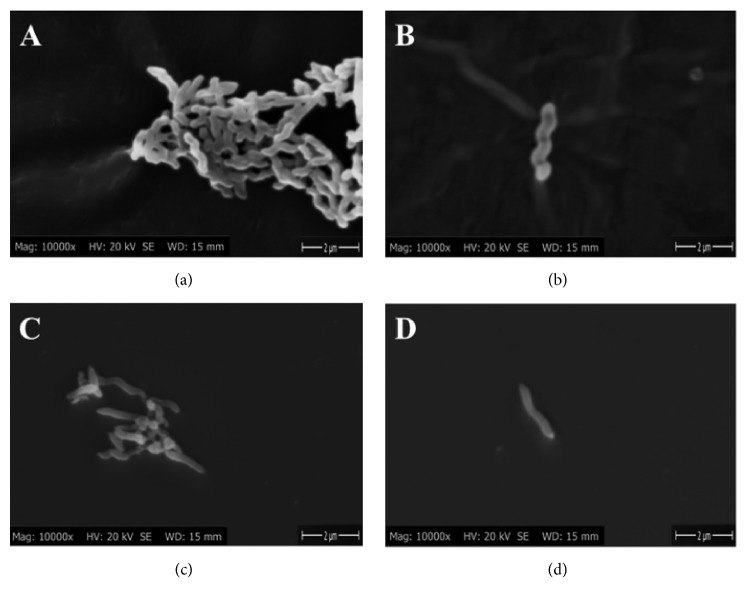
**Scanning electron micrographs of nontreated (a, b) and PEO treated (150** ***μ*** **g mL** ^**-1**^ **) (c, d)* C. jejuni *NCTC 11168 cells.** Instrumental magnification was 10,000x.

Scanning electron microscopy was performed to reveal the effect of PEO on the cell morphology of* C. jejuni*. Of the nontreated cells, 80% possessed a distinct spiral form (Figures 2(a) and 2(b)) characteristic of* C. jejuni*. Some cells (20%) lost their typical spiral shape.

The most striking effect came after 30 min PEO treatment, where 63% of cells became straight (Figures 2(c) and 2(d)). Only 12% had the typical spiral shape, and 25% showed a transient shape. At the applied concentration (150 *μ*g mL^−1^) we have found no coccoid forms as described by other authors in the presence of other environmental stresses [31].

### 3.3. Effect of PEO on the Motility of* C. jejuni*

**Figure 3:**
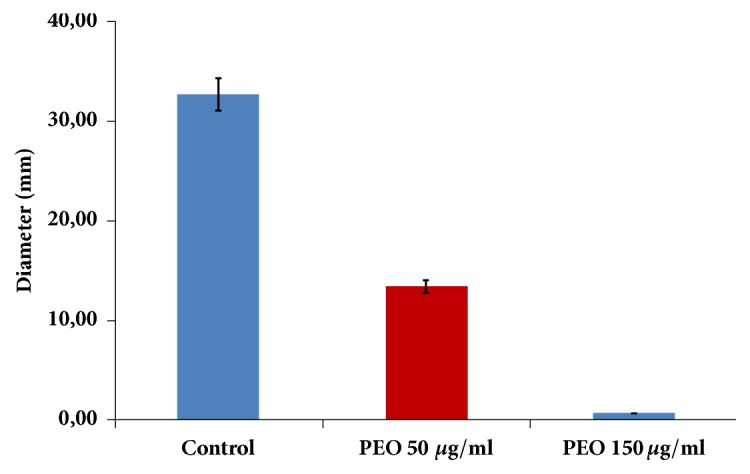
**Soft agar swarming assay of the* C. jejuni* NCTC 11168 in the presence and lack of PEO at 37**°**C. **Without PEO, cells (column A) show strong swarming activity in the 0.3% agar plates, while this feature was impaired in the otherwise sublethal 50 *μ*g mL^−1^ PEO concentration (column B). No turbidity was observed around the inoculation site in the plates having a 150 *μ*g mL^−1^ PEO concentration. Plates were incubated for 24 h.

The motility assay of untreated and treated* C. jejuni* revealed that PEO can inhibit the spread of the bacteria. After 24 h microaerobic incubation, untreated cells had a 32.67 ± 3.21 mm turbid area (Figure 3), decreasing markedly in the* C. jejuni* cells treated with 50 *μ*g mL^−1^ PEO (13.33 ± 4.04 mm; Figure 3). No swarming was observed if cells were inoculated into the soft agar medium containing 150 *μ*g mL^−1^ PEO (Figure 3). Motility assays were performed 3 times and the average diameters are presented (Figure 3).

### 3.4. Gene Expression Profile of* C. jejuni* in Response to PEO

To understand the molecular basis of the impact of PEO treatment on* C. jejuni* cells, 44 genes were analyzed by qRT-PCR (Supplementary Table). General stress genes showed the most marked changes (Table 1). Except for* ahpC* (7.18x) involved in oxidative stress response [30], the dominance of a general stress response was found:* dnaK *(10.41x) [26],* groEL *(3.63x), and* groES *(4.77x) [25, 39]. Some of the investigated virulence-associated factors were downregulated while others showed a marked expression compared to that of the control. From the 3 known sigma factors,* rpoN* and* rpo*D were downregulated, in contrast to* fliA* which was upregulated by a factor of 1.7, respectively.

### 3.5. PEO Induced Changes in Proteome

In order to identify the proteins affected by PEO, 10% 2D polyacrylamide gel electrophoresis was used. Bands that showed marked elevation or decrease in 3 parallel 2D SDS-PAGE runs were analyzed by LC-MS. Two protein bands had a decreased expression level compared to the control sample (Table 2). These bands correspond to proteins involved in the synthesis of two virulence-associated factors: PEB4 [6], a temperature dependent colonization factor, and HtrA, a serine protease with a role in adherence and invasion [40]. Additionally 10 proteins had an elevated expression level compared to the control (Table 2), including 2 adhesion factors (PEB1 and PEB3) [41], a stress response protein (DnaK) [26], an elongation factor (Tu) [42]. Elevated expression levels were detected for oxidoreductase [43], and adenylate kinase [44], enzymes with roles in energy metabolism, succinyl CoA, and thiol peroxidase [45], an oxidative stress response protein (Table 2).

### 3.6. Identification of PEO Components with Antibacterial Activity

**Figure 4:**
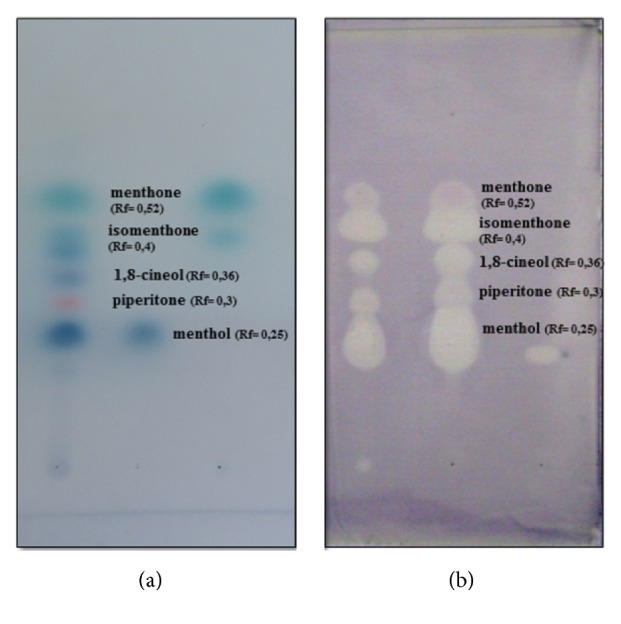
**Thin layer chromatography separation (a) and antibacterial effect of PEO components (b).** TLC separated PEO components ((a)/1^st^ column) were visualized by ethanolic vanillin–sulphuric acid reagent in the presence of reference compounds menthol ((a)/2^nd^ column), menthone, and isomenthone ((a)/3^rd^ column). Antibacterial effect of separated PEO components was revealed by bioautography (b). Sample application: (a)/1^st^ and (b) column, 0.2 *μ*l of PEO (100 *μ*l/500 *μ*l); (a)/2^nd^, 1 *μ*l of menthol standard (10 mg/mL); (a)/3^rd^, 1 *μ*l of menthone (10 mg/mL) and 1 *μ*l of isomenthone (10 mg/mL) standards.

Compound composition of PEO was determined by GC-MS analyses (Table 3). By using TLC plates with alcoholic vanillin–sulphuric acid reagent, 8 components were obtained (Figure 4(a)). Three main components (menthone at R_f_ = 0.52, menthol R_f_ = 0.25, and isomenthone R_f_ = 0.4) were detected by applying standard reference compounds. Piperitone R_f_ = 0.3 and 1,8-cineol R_f_ = 0.36 were identified by their R_f_ values [46]. Direct bioautography reveals that at least 7 different components of PEO have an antimicrobial effect on* C. jejuni *(Figure 4). No clearing zone was detected in the case of the sole solvent control.

## 4. Discussion

Peppermint (*Mentha piperita*) is one of the oldest and most highly regarded herbs for treating gastrointestinal diseases [16]. As* Campylobacter jejuni* is the most frequently reported food-borne pathogen in human gastrointestinal infections worldwide [1, 2] and the anti-*Campylobacter* effect of peppermint essential oil (PEO) on two isolates was previously reported [18, 19], we confirmed the anti-*Campylobacter* effect of PEO on a broader strain collection for the first time. A well-characterized* C. jejuni* collection, isolated from hospitalized patients [9] and 4 widely studied reference strains, was used. The* C. jejuni* strain NCTC 11168 was used for the more detailed analysis and experiments were carried out between the MIC and MBC values (100 *μ*g mL^−1^ and 400 *μ*g mL^−1^, respectively) in the sublethal range, as this concentration range is ideal for stress response studies in bacteria [47, 48]. In our case we have chosen the 150 *μ*g mL^−1^ PEO concentration and 10-minute exposition time as this concentration was in the sublethal range and at this timepoint the bacterial cell count was only decreased to 99.5% compared to the starting CFU (Figure 1). In pilot it was also revealed that this condition has already induced clearly detectable transcriptomic and proteomic changes.

In order to reveal the expression changes of the most important virulence-associated genes, we have investigated 23 target genes. These genes are known to be clearly associated with virulence and were used in one of our previous studies [15]. The varying degrees of influence of this 23 investigated virulence-associated genes suggest that PEO could have an effect on different targets and regulatory routes. The drastic suppression (0,08x) of* cadF* and* cbf2* (PEB4A) [6], 2 characterized adhesive proteins of* C. jejuni*, correlates with recent findings where antiadhesive effects of different extracts of* C. jejuni* on different surfaces were investigated [14, 49]. On the other hand, the increased expression of PEB1 [5] and PEB3 [41] (Table 2.), 2 proteins also thought to be involved in adhesion, suggests that PEO might also increase the expression of certain virulence-associated genes.

Impaired motility was a clear demonstration that one of the most important virulence traits of* C. jejuni* was affected by PEO. The molecular background of this phenomenon raises questions, as this outcome is based on the function of a complex machinery consisting of nearly 40 genes [50]. The targeted investigation of both structural and regulatory genes from this system has revealed that despite the upregulation of* flaB* (3.6x)-coding for the subunit protein of the filament,* flgB* (1.93x)-coding for flagellar basal body protein, and* flgE2* (2.0x)-coding for the flagellar hook, motility was impaired. The subtle network of these genes is under the control of the three global regulators in* C. jejuni*: *σ*-70 (RpoD), *σ*-54 (RpoN), and *σ*-28 (FliA) [51]. Impaired motility with the slight but steady suppression of* rpoN* (0.72x) correlates with earlier findings [52] where* rpoN* mutants lost their motility. Decreased expression influences both structural and chemotactic genes [52] that we also demonstrated with the suppression of* flhB* (0.79x),* cheY* (0.84x), and* docB* (0.75x).

Treatment with antimicrobial agents is an environmental stress stimulus for bacteria. One typical feature of the* C. jejuni* stress response is that cells are prone to fall into a viable but nonculturable (VBNC) state, characterized by rounded cell morphology [31, 32]. Starvation [53], heat [54], or oxidative stresses [55] also lead to this cell morphology. The straightened cell morphology observed here (Figure 2) upon PEO treatment was formerly unreported in wild type strains, although it was observed in* rpoN* mutants [56]. Therefore* rpoN* suppression not only impedes motility, but also affects cell morphology embodied in a linearized, elongated form. The unique stress response induced by PEO treatment differs from another recently studied EO, clove (CEO), that in contrast induces the shortening of* C. jejuni* cells [15].

This observation suggested that there is a difference between the two dominant stress responses evoked by PEO and CEO. The constant expression of the three most important genes (*dps*,* sodB*, and* katA*) involved in oxidative stress responses [31] suggested that cytoplasmic membrane disruption and leakage were not the major antibacterial mode of action of PEO, as was suggested for other EOs [21–23]. The marked increase (3.63x, 4.77x, and 10.41x) in the transcription of the three important molecular chaperones (*groEL*,* groES*, and* dnaK)* indicated the dominance of a nonoxidative stress response [25, 26]. These three enzymes belong to the heat stress protein family, but have essential roles in osmotic [57] and possibly general stress responses [25, 26].

The markedly elevated level of* dnaK*/DnaK (Tables 1 and 2) supports the chemical nature of the stress response [26]. This also supports recent findings [25] that argue against the role of* groEL* (Table 1) in an oxidative stress response. The (i) moderate expression levels of* dps*,* sodB*, and* katA,* (ii) elevated levels of* groEL*,* dnaK*, and* groES,* and (iii) elongated cell morphology all indicate that PEO does not primarily induce oxidative stress. On the other hand the elevated expression levels of two alkyl hydroperoxide reductases, AhpC and thiol peroxidase (Table 2: 8, 11), are however seemingly contradictory. AhpC from* C. jejuni* was originally described in oxidative stress conditions [58] and recent investigations revealed that it detoxifies the cell from reactive oxygen species (ROS) and lipid hydroperoxides (LPOs) and is involved in biofilm formation [59, 60]. Genes involved in general and oxidative stress responses are often induced in biofilms, possibly to alleviate the stress generated under such conditions [61, 62].

On the other hand AhpC is associated with resistance to the antituberculotic drug isoniazid (INH) in* Mycobacterium tuberculosis* [63]. Based on these and our results, we cannot exclude the direct or indirect detoxification role of AhpC and thiol reductase in* C. jejuni*. It might also repair damaged molecules during general or chemical stresses or simply inactivate toxic molecules explaining the more than 7-fold increase in expression of* ahpC *by RT-PCR (Table 1). The comparable high expression (8.04x) of* cmeB* also indicates the chemical characteristics of the PEO stress response as RND family transporters are active efflux systems of many antibiotics and chemicals mostly in Gram negative microorganisms [64].

Stress conditions cause a revved metabolism, which is indicated by elevated energy traffic through the higher expression of three typical enzymes (oxidoreductase, adenylate kinase, and succinyl CoA) (Table 2). The stabilization of basic metabolism, a matter of life or death during stress conditions, depends on fast and adequate responses. The unchanged expression levels of all 12 investigated housekeeping genes indicate that components of PEO in the investigated concentration do not express their antimicrobial effect by interfering with basic metabolism.* Pgm*, encoding phosphoglucosamine mutase, was the most stable and was applied as an internal control to unequivocally define and evaluate the transcriptomic activity of the tested genes. Maintenance of basic metabolism during stress conditions is crucial, and transcription and elongation factors (EL) bear considerable burden. EL-TU is one of the three prokaryotic ELs and has a role in fast and precise synthesis of proteins [42]. Its pivotal supportive role in efficient protein folding controlled by* groEL* (Tables 1 and 2) and its lid-like chaperonin protein complex GroES [39] was demonstrated.

In this study, we showed that PEO exhibited general anti-*Campylobacter* activity whose effect could be dedicated at least to 5 out of the 9 compounds. During this exposure, the characteristic feature of the evoked stress response was more similar to a general stress response rather than an oxidative one. The response observed was characterized by impaired ability to swarm, downregulation of certain virulence-associated genes, and elongated cell morphology, in contrast to the rounded cell morphology typically observed under oxidative stress conditions. On the contrary, we found that some virulence-associated genes were upregulated. The challenge of future studies will be to identify those individual PEO compounds that can selectively hinder the expression of a repertoire of virulence-associated genes or specifically block different cellular mechanisms. Identification of potential compounds will contribute to the control of this important food-borne pathogen where occurrence of both hypervirulent [10] and multidrug resistant clones [11] from animal husbandries was recently reported.

## Figures and Tables

**Table 1:** Transcriptional intensity changes of the most affected stress related ORFs and the three global regulators of *C. jejuni* NCTC 11168 in the presence of PEO. The table represents genes with significantly altered expression level, p<0.001. The applied PEO concentration was 150 *µ*g mL^−1^.

*C. jejuni* 11168 ORF	Gene name	Fold change	Specific function
Cj0334	*ahpC*	7.18±0.33	Alkyl hydroperoxide reductase
Cj1221	*groEL*	3.63±0.81	Molecular chaperone GroEL
Cj0759	*dnaK*	10.41±0.4	Molecular chaperone DnaK
Cj1220	*groES*	4.77±0.54	Co-chaperonin GroES
Cj0061c	*fliA*	1.71±0.04	Sigma factor (Flagellar biosynthesis)
Cj0670	*rpoN*	0.72±0.07	RNA polymerase factor sigma-54
Cj1001	*rpoD*	0.11±0.05	RNA polymerase factor sigma-70

**Table 2:** Proteins identified by SDS-PAGE separation (pH3.0–10.0 gradient gels) followed by in-gel digestion and LC-MS analysis. Last column (Expression) represents the state of proteins upon PEO treatment. The applied PEO concentration was 150 *µ*g mL^−1^.

Spot No.	Protein name	Mascot Score	Molecular weight [kDa]	Peptides	UniProt/Accession. Number	Expression (rate of change)	Confirmation by RT-PCR (fold change)
1	Serine protease htrA	555.0	50.9	15.0	gi|218562840	-3x	0.47
2	Major antigen cbf2/PEB4A	612.5	30.4	23.0	CBF2_CAMJE	-8x	0.23
3	Elongation factor Tu	682.6	43.5	28.0	EFTU_CAMJD	+4x	2.82
4	Uncharacterized protein	463.7	20.9	15.0	gi|121612795	+6.5x	5.75
5	Major cell-binding factor PEB1	343.8	28.1	12.0	PEB1A_CAMJE	+4x	7.10
6	Succinyl-coA synthetase alpha chain	744.7	30.0	23.0	gi|384447814	+4x	2.14
7	Major antigenic peptide PEB3	137.2	27. 5	6.0	gi|57237344	+1.5x	1.43
8	Anti-oxidant AhpC/TSA family protein	446.5	21.9	13.0	gi|57237385	+4x	3.84
9	Oxidoreductase subunit	374.8	26.9	16.0	gi|121613212	+2x	1.98
10	Adenylate kinase OS	337.5	21.3	11.0	KAD_CAMJE	+3x	2.28
11	Probable thiol peroxidase OS	702.7	18.4	23.0	TPX_CAMJE	+3x	2.56
12	Molecular chaperone DnaK	366.0	67.3	15.0	gi|57237604	+5x	10.41

**Table 3:** Volatile compound composition of PEO determined by GC-MS.

****	**Name of** **compounds**	**t**_**R**_ **MS**	**t**_**R**_ ** FID**	**Incidence ** ** (**%**)**
		(min)	(min)	
1	*α*-pinene		5.8	1.1
2	*β*-pinene	6.9	6.7	0.6
3	Limonene		7.4	1.4
4	p-cymol		7.6	0.2
5	1,8 cineole	8.0	7.9	5.5
6	Isopulegone	10.5	11.1	1.0
7	Menthone	10.6	11.2	19.8
8	Isomenthone	10.8	11.5	7.0
9	Isomenthol	10.9	11.6	4.3
10	Menthol	11.1	11.9	50.4
11	Menthyl-acetate	13.1	13.0	5.5
12	Piperitone	12.6	13.8	0.8
13	*β*-caryophyllene	15.4	14.8	0.4
14	Caryophyllene oxide	18.0	18.8	0.1

## Data Availability

All data used to support the findings of this study are included within the article and in the Supplementary Materials of the paper.

## Supplementary Materials

Supplementary Materials Supplementary Table: genes and primers selected for RT-PCR. Primer sequences were designed in this study.
