# Supplementary material for: Stress Response and Virulence Potential Modulating Effect of Peppermint Essential Oil in Campylobacter jejuni
Source: Biomed Res Int. 2019 Jan 3;2019:2971741. doi: 10.1155/2019/2971741 (PMC6335803; doi:10.1155/2019/2971741)
Supplement: Supplementary Materials — Supplementary Table: genes and primers selected for RT-PCR. Primer sequences were designed in this study. [file 2971741.f1.docx]

**Supplementary Table**

Genes and primers selected for RT-PCR. Primer sequences were designed in this study. Fold change 1,00 equals with the untreated sample. Higher fold changes mean higher expressions, while lower fold changes mean lower expression levels. The housekeeping gene [phosphoglucosamine mutase](http://www.ncbi.nlm.nih.gov/nucleotide/408667903?report=gbwithparts&amp;from=326929&amp;to=328266&amp;RID=9MEVDFGP01R) (*pgm*) was used as internal control throughout the experiments.

| Gene function | | Gene | | Primer | | Sequence(5'-3') | | Fold change | |  | |
| --- | --- | --- | --- | --- | --- | --- | --- | --- | --- | --- | --- |
| **Oxidative stress response genes Peroxide sensing regulator** | |  | |  | |  | |  | |  | |
| DNA protection during starvation protein | *dps* | | Fw | | CTCTATTGTCTTGATTAGGA | | 1,585±0,01 | |  | |  |
|  |  | | Rev | | AATCTTGCGACAAATCCAAA | |  | |  | |  |
| superoxide dismutase | *sodB* | | Fw | | TGGCGGTTCATGTCAAAGTA | | 1,55±0,04 | |  | |  |
|  |  | | Rev | | ACCAAAACCATCCTGAACCA | |  | |  | |  |
| alkyl hydroperoxide reductase | *ahpC* | | Fw | | CTTGCTTGATGCTGATGGAA | | 7,185±0,33 | |  | |  |
|  |  | | Rev | | GGGTTAGCTTTCATACCTTC | |  | |  | |  |
| catalase | *katA* | | Fw | | ATCCTGATGCACAAAGATAT | | 1,852±0,12 | |  | |  |
|  |  | | Rev | | GACTAAATCAGGTTCAAGAT | |  | |  | |  |
| **General stress response genes** |  | |  | |  | |  | |  | |  |
| co-chaperonin | *groES* | | Fw | | AACCTTTAGGAAAGCGTGTT | | 4,77±0,54 | |  | |  |
|  |  | | Rev | | CACCATTTGCAATATCAGTG | |  | |  | |  |
| molecular chaperone | *groEL* | | Fw | | CTATGCTTGAAGATATAGCG | | 3,625±0,81 | |  | |  |
|  |  | | Rev | | CTAGAAGCTTGTCCAAGATC | |  | |  | |  |
| chaperone | *dnaK* | | Fw | | CTCTACTCGTGTTCCTTTAGT | | 10,41±0,4 | |  | |  |
|  |  | | Rev | | CTAGCATCTACAGCTTCTTTG | |  | |  | |  |
|  |  | |  | |  | |  | |  | |  |
| **Virulence factors** |  | |  | |  | |  | |  | |  |
| chemotaxis regulatory protein | *cheY* | | Fw | | AACTTGTGGAGTAAAAGGTT | | 0,845±0,023 | |  | |  |
|  |  | | Rev | | AGATTGGAATATGCCAGAAA | |  | |  | |  |
| flagellin | *flaB* | | Fw | | CATATCAGCATTGATAAAGG | | 3,605±0,45 | |  | |  |
|  |  | | Rev | | TATGAAGATGGTGATGGAAA | |  | |  | |  |
| flagellar biosynthesis protein | *flhB* | | Fw | | AATTTATGCAAGAGCTTCCA | | 0,79±0,07 | |  | |  |
|  |  | | v | | TACGAATTCTACCTTTAACC | |  | |  | |  |
| flagellar basal body rod protein | *flgB* | | Fw | | AGAGCTGTTATCATAACTGT | | 1,935±0,31 | |  | |  |
|  |  | | Rev | | TGGTAAATCGTGCAAATGAA | |  | |  | |  |
| outer membrane fibronectin-binding protein | *cadF* | | Fw | | TCAAGTTCATTAGCAACACT | | 0,08±0,003 | |  | |  |
|  |  | | Rev | | TTGGAAGGTCATTTTGGTTT | |  | |  | |  |
| periplasmic cytochrome C peroxidase | *docA* | | Fw | | TAAATCTTCTTGGTTTGGGT | | 1,06±0,03 | |  | |  |
|  |  | | Rev | | TGCAGTTGCTGAATTTGAAA | |  | |  | |  |
| methyl-accepting chemotaxis protein (MCPs) | *docB* | | Fw | | GTTGTCATTGATTTGTTGGA | | 0,75±0,082 | |  | |  |
|  |  | | Rev | | CCAAAGTGCGGATAATATTA | |  | |  | |  |
| methyl-accepting chemotaxis protein (MCPs) | *docC* | | Fw | | AAGGCAAGAAATTCTTTAGC | | 0,92±0,043 | |  | |  |
|  |  | | Rev | | CCATTTGGTTGAGCAATATA | |  | |  | |  |
| cytolethal distending toxin | *cdtB* | | Fw | | TCATTTCCATTGCGAATTCC | | 0,945±0,011 | |  | |  |
|  |  | | Rev | | GGAATTTAGGAACTCTTTC | |  | |  | |  |
| β-1,3 galactosyltransferases to synthesize ganglioside mimics | *wlaN* | | Fw | | TGCTGGGTATACAAAGGTTGTG | | 3,575±0,37 | |  | |  |
|  |  | | Rev | | AATTTTGGATATGGGTGGGG | |  | |  | |  |
| flagellar hook protein | *flgE2* | | Fw | | CATCTCACCACGACCTCCTGTTC | | 0,205±0,47 | |  | |  |
|  |  | | Rev | | GCAAAAATCGCAATGGCTTCA | |  | |  | |  |
| **LPS sytnhesis genes** |  | |  | |  | |  | |  | |  |
| UDP-glucose 4-epimerase | *galE* | | Fw | | GCTTCAATCACTTCTTTTAC | | 0,895±0,031 | |  | |  |
|  |  | | Rev | | TTTGGCGATGATTATGATAC | |  | |  | |  |
| ABC transporter, permease/ATP-binding protein | *wlaB* | | Fw | | ATCCATGATTTTTGCTTCAC | | 0,89±0,029 | |  | |  |
|  |  | | Rev | | AATATCACTTTTGGAGATGC | |  | |  | |  |
|  |  | |  | |  | |  | |  | |  |

| **Putative virulence genes** | | | |  | | | |  |  | | |  | |  | |
| --- | --- | --- | --- | --- | --- | --- | --- | --- | --- | --- | --- | --- | --- | --- | --- |
| oxidoreductase | *Cj0415* | Fw | | | | CCTTGTCAATACTGTGCGTAT | | | | 1,515±0,07 | | |  | |  |
|  |  | Rev | | | | GTTCCAAGCTGAACGCTATAA | | | |  | | |  | |  |
| D-3-phosphoglycerate dehydrogenase | *serA* | Fw | | | | CCTAAAATTCCACCCAAAGCA | | | | 0,48±0,009 | | |  | |  |
|  |  | Rev | | | | GATTGTACACAGAAGAGGCTT | | | |  | | |  | |  |
| quinone-reactive Ni/Fe-hydrogenase, large | *hydB* | Fw | | | | ATCGATCACATAAGGAGCACA | | | | 3,85±0,31 | | |  | |  |
|  |  | Rev | | | | AATCGGAAGAGATGAATGGCT | | | |  | | |  | |  |
| RND efflux system, inner membrane transporter | *cmeB* | Fw | | | | CCATCTTTCATATTGGGCAG | | | | 8,04±0,75 | | |  | |  |
|  |  | Rev | | | | GATGAATGCAACTATAGGCAC | | | |  | | |  | |  |
| major outer membrane protein | *porA* | Fw | | | | CAGTGCTGCTATAGCTGATAA | | | | 0,25±0,011 | | |  | |  |
|  |  | Rev | | | | CCTAAGTAAGCACCTTCAAGT | | | |  | | |  | |  |
| 2-oxoglutarate-acceptor oxidoreductase subunit | *oorA* | Fw | | | | CCTATTACTCCTAGTAGTGAG | | | | 2,901±0,093 | | |  | |  |
|  |  | Rev | | | | CCATGATGAAGTCCTGTAACA | | | |  | | |  | |  |
| methyl-accepting chemotaxis signal transduction protein | *Cj1564* | Fw | | | | CGATCTCCATCTTGAGTAAGA | | | | 0,78±0,04 | | |  | |  |
|  |  | Rev | | | | CTGCTAATCGTTCTATGGCTA | | | |  | | |  | |  |
| sucC succinyl-CoA synthase, beta subunit | *sucC* | Fw | | | | GCTATCCTATCACAACGAACA | | | | 0,615±0,078 | | |  | |  |
|  |  | Rev | | | | GGACTTGAAGTAGCGAGAGTT | | | |  | | |  | |  |
| thiamine biosynthesis protein | *thiC* | Fw | | | | GCTATGATAGGAACAGTTCCT | | | | 1,42±0,045 | | |  | |  |
|  |  | Rev | | | | CCTGGACCTTCTATCATTACT | | | |  | | |  | |  |
| putative transmembrane transport protein | *C8J_* | Fw | | | | AGCTTGTGCATCTGCTTCTAT | | | | 1,025±0,02 | | |  | |  |
|  | 1184 | Rev | | | | ATCGATCCAGTTGTTCGTGAT | | | |  | | |  | |  |
| **Housekeeping genes** |  |  | | | |  | | | |  | | |  | |  |
| hippurate hydrolase | *hipO* | Fw | | | | ATCTAATGCTCTAACACTCA | | | | 0,703±0,06 | | |  | |  |
|  |  | Rev | | | | GCAAAAGATCCTATTTATGC | | | |  | | |  | |  |
| [glutamine synthetase](http://www.ncbi.nlm.nih.gov/nucleotide/408667903?report=gbwithparts&amp;from=658489&amp;to=659919&amp;RID=9MH678K001R) | *gln* | Fw | | | | TTCATTTTCTGGTCCAAAGT | | | | 2,685±0,12 | | |  | |  |
|  |  | Rev | | | | CTGATCCTACTATCATAGTA | | | |  | | |  | |  |
| [citrate synthase](http://www.ncbi.nlm.nih.gov/nucleotide/408667903?report=gbwithparts&amp;from=1598392&amp;to=1599660&amp;RID=9MH49YPC01R) | *glt* | Fw | | | | GCATACCTTCATGGATAAAA | | | | 0,24±0,04 | | |  | |  |
|  |  | Rev | | | | ATGTTTTCTTATGATGAGGG | | | |  | | |  | |  |
| [transketolase](http://www.ncbi.nlm.nih.gov/nucleotide/408667903?report=gbwithparts&amp;from=1563521&amp;to=1565419&amp;RID=9ME1WJ1P014) | *tkt* | Fw | | | | TCAACTCTTGGAGTAGAAAT | | | | 0,765±0,067 | | |  | |  |
|  |  | Rev | | | | GCTAAAGAACAAGCTTCATA | | | |  | | |  | |  |
| [phosphoglucosamine mutase](http://www.ncbi.nlm.nih.gov/nucleotide/408667903?report=gbwithparts&amp;from=326929&amp;to=328266&amp;RID=9MEVDFGP01R) | *pgm* | Fw | | | | GGAAAAGATACAAGAAGAAG | | | | 1,000±0,025 | | |  | |  |
|  |  | Rev | | | | CAACGCATATCTTCAGTTAA | | | |  | | |  | |  |
| [F0F1 ATP synthase subunit alpha](http://www.ncbi.nlm.nih.gov/nucleotide/408667903?report=gbwithparts&amp;from=111953&amp;to=113458&amp;RID=9MGNM6KF016) | *uncA* | Fw | | | | GTAGGTATTGTTATACTTGG | | | | 0,84±0,052 | | |  | |  |
|  |  | Rev | | | | TTCATTAGCATTGATCACAC | | | |  | | |  | |  |
| [DNA gyrase subunit A](http://www.ncbi.nlm.nih.gov/nucleotide/408667903?report=gbwithparts&amp;from=958155&amp;to=960746&amp;RID=9MUGVKV4014) | *gyrA* | Fw | | | | CATCATAAACTGCTGTATCT | | | | 1,45±0,073 | | |  | |  |
|  |  | Rev | | | | GTTATTATAGGTCGTGCTTT | | | |  | | |  | |  |
| ketol-acid reductoisomerase | *ilvC* | Fw | | | | GGTAGTGTAAGTGCTGTTAA | | | | 0,64±0,002 | | |  | |  |
|  |  | Rev | | | | TGAATTTCATCAGGAGCTAA | | | |  | | |  | |  |
| [DNA-directed RNA polymerase subunit alpha](http://www.ncbi.nlm.nih.gov/nucleotide/408667903?report=gbwithparts&amp;from=1518228&amp;to=1519241&amp;RID=9MVA81YD01R) | *rpoA* | Fw | | | | GCCAACAGAATTTACAATAG | | | | 0,435±0,072 | | |  | |  |
|  |  | Rev | | | | AAGTGCTACATCTTCAAGCA | | | |  | | |  | |  |
| [FKBP-type peptidyl-prolyl cis-trans isomerase SlyD](http://www.ncbi.nlm.nih.gov/nucleotide/408667903?report=gbwithparts&amp;from=120659&amp;to=121228&amp;RID=9N1TRPM501R) | *slyD* | Fw | | | | AAAGCTTAGAAGAAGAAGTG | | | | 0,5±0,049 | | |  | |  |
|  |  | Rev | | | | AATACCTGCAAATTGCTCTT | | | |  | | |  | |  |
| [rRNA-16S ribosomal RNA](http://www.ncbi.nlm.nih.gov/nucleotide/408667903?report=gbwithparts&amp;from=37510&amp;to=39023&amp;RID=9N1JWFGU014) | *rrs* | Fw | | | | AAATCCGTAGATATCACCAA | | | | 1,83±0,107 | | |  | |  |
|  |  | Rev | | | | TAGCTGCATTACTGAGATGA | | | |  | | |  | |  |
| **Transcription regulator** |  |  | | | |  | | | |  | | |  | |  |
| RNA polymerase factor sigma-70 | *rpoD* | Fw | | | | TTTAGAATACCGCTTACCTA | | | | 0,11±0,05 | | |  | |  |
|  |  | Rev | | | | GCGTTTAATTTGCTCCAAAA | | | |  | | |  | |  |
| RNA polymerase factor sigma-54 | *rpoN* | Fw | | | | GGAGTTAGATGAAGATATTG | | | | 0,72±0,067 | | |  | |  |
|  |  | Rev | | | | CCATCAGTTTGGATTGAAAT | | | |  | | |  | |  |
| flagellar biosynthesis factor sigma-28 | *fliA* | Fw | | | | GCCTTCAAGTATAGATGTTA | | | | 1,71±0,045 | | |  | |  |
|  | | | Rev | |  | | TCCATGATTGCATCAATATC | | | |  |  |  |  |  |
